# A novel rapid and reproducible flow cytometric method for optimization of transfection efficiency in cells

**DOI:** 10.1371/journal.pone.0182941

**Published:** 2017-09-01

**Authors:** Stefanie Homann, Christian Hofmann, Aleksandr M. Gorin, Huy Cong Xuan Nguyen, Diana Huynh, Phillip Hamid, Neil Maithel, Vahe Yacoubian, Wenli Mu, Athanasios Kossyvakis, Shubhendu Sen Roy, Otto Orlean Yang, Theodoros Kelesidis

**Affiliations:** David Geffen School of Medicine at University of California Los Angeles, Los Angeles, United States of America; Augusta University Medical College of Georgia, UNITED STATES

## Abstract

Transfection is one of the most frequently used techniques in molecular biology that is also applicable for gene therapy studies in humans. One of the biggest challenges to investigate the protein function and interaction in gene therapy studies is to have reliable monospecific detection reagents, particularly antibodies, for all human gene products. Thus, a reliable method that can optimize transfection efficiency based on not only expression of the target protein of interest but also the uptake of the nucleic acid plasmid, can be an important tool in molecular biology. Here, we present a simple, rapid and robust flow cytometric method that can be used as a tool to optimize transfection efficiency at the single cell level while overcoming limitations of prior established methods that quantify transfection efficiency. By using optimized ratios of transfection reagent and a nucleic acid (DNA or RNA) vector directly labeled with a fluorochrome, this method can be used as a tool to simultaneously quantify cellular toxicity of different transfection reagents, the amount of nucleic acid plasmid that cells have taken up during transfection as well as the amount of the encoded expressed protein. Finally, we demonstrate that this method is reproducible, can be standardized and can reliably and rapidly quantify transfection efficiency, reducing assay costs and increasing throughput while increasing data robustness.

## Introduction

Transfection is one of the most common used techniques in molecular biology [1, 2]. Transfection is the process of introducing plasmid nucleic acid (DNA that carries a gene of interest or mRNA) into target cells that then eventually express the desired nucleic acid or protein. There are a number of strategies for introducing nucleic acids into cells that use various biological, chemical, and physical methods [1–3]. However, there is a wide variation with respect to transfection efficiency, cell toxicity, the level of gene expression, etc. To determine how these factors influence transfection, a sensitive and robust detection assay is required to quantify and optimize the efficiency of different transfection methods to deliver the target gene into the cytosol and facilitate protein expression while reducing cell toxicity.

Researchers often use easily tractable reporter assays for determining transfection efficiency and their downstream applications [1, 2]. Commonly used reporters include firefly or renilla luciferase and the green fluorescent protein (GFP). The luciferase assay is sensitive and suitable for determining relative transfection performance between samples but has several limitations since it requires cell lysis and does not quantify cell toxicity of the transfection method [4]. Cells expressing the GFP reporter can be visualized directly by fluorescence microscopy, which can be subjective, and laborious [5]. Flow cytometry is excellent/the state of the art for quantitative phenotyping in a large population of cells with high sensitivity, can be combined with cell sorting for downstream applications [6] and represents the most accurate and objective method for determining transfection efficiency [6], monitoring expression of inducible reporters [7] and for detecting time-dependent degradation of target proteins [8]. Most recent flow cytometric methods to quantify transfection efficiency in cells are based on transfection of GFP-fusion proteins or co-transfection of GFP plasmids. Both strategies have their limitations including competition in expression of the two different plasmids that can compromise transfection efficiency of the plasmid of interest [9, 10], unequal delivery of plasmids between cells that may affect linearity of reporter expression [6, 9–11], inconsistent transfection based on the type of reporter plasmid that can introduce significant experimental bias in estimation of transfection efficiency [12, 13] and artifacts of GFP fluorescence during processing of cells or tissues [14, 15]. Most importantly, we do not know the exact nature of the interaction between different co-transfected reporter genes that causes variation in their activities [12, 13].

An alternative and more direct method to using fluorescent reporter genes is to directly label nucleic acids with fluorescent dyes to track their intracellular delivery [16]. Non-radioactive enzymatic labeling methods are inherently difficult to control and generate labeled products that are not representative of the starting DNA [17]. Using the non-enzymatic Label IT^®^ Tracker TM Kits, any plasmid can be custom labeled in a simple one-step chemical reaction before introduction into mammalian cells [18]. Thus, both subcellular localization of the labeled DNA and expression reporter transgene can be monitored simultaneously following introduction of the labeled plasmid into mammalian cells [16, 18]. This method has previously been used for immunofluorescence experiments, however, as mentioned above, this approach can be subjective, qualitative, and laborious [5, 16, 18].

Herein, we demonstrate the development of a flow-cytometric assay to determine transfection efficiency by labeling a reporter plasmid with Label IT^®^ TrackerTM. This method does not depend on co-transfection of two different plasmids and simultaneously quantifies cell death, uptake of the labeled plasmid during transient transfection, and expression of the target protein. We demonstrate that this method can be used as a tool to i) optimize transfection efficiency in a standard cell line ii) to quantify cellular toxicity of different transfection methods iii) to determine uptake of DNA into difficult to transfect cells via electroporation without the need to use co-transfection of GFP plasmid that can further reduce the efficiency of transfection. This flow cytometric method can be directly applied to optimize several transfection methods including gene therapy strategies (e.g. CRISPR/Cas system).

## Materials and methods

### Cells

293T cells were maintained with Dulbecco's modified Eagle medium (DMEM) (Invitrogen, Carlsbad, CA) supplemented with 10% fetal bovine serum (FBS) (Omega Scientific, Tarzana, CA) and penicillin and streptomycin (Invitrogen). Jurkat E6-1 (obtained through the NIH AIDS Reagent Program, Division of AIDS, NIAID, NIH: Jurkat Clone E6-1 from Dr. Arthur Weiss)[19] were cultured in RPMI 1640 medium (Invitrogen) supplemented with 10% fetal bovine serum (FBS), L-glutamine, penicillin, and streptomycin. All cells were incubated at 37°C and 5% CO_2_.

### Plasmids

pNL4-3 is a full-length, replication and infection competent chimeric HIV DNA plasmid (14825 bp) that was received from AIDS reagent program (#114, NIH)[20]. pUltraHot encoding mCherry (8314 bp) was a kind gift of Jeff F. Miller, California NanoSystems Institute.

### Nucleic acid labeling of plasmids

Plasmid DNA for transfection was extracted from Stbl3^™^ (Thermo Fisher, Waltham, MA) or Stellar (Clontech, Mountain View, CA) bacteria using the PureLink HiPure Midiprep Kit (Thermo Fisher, Waltham, MA). The concentration was determined using spectrophotometry and the purity was between 1.8–2.0 (260/280 ratio). Plasmid DNA was labeled with DNA LabelIT Tracker (Mirus, Madison, WI) the day before transfection using 0.5 ul FITC/1 μg DNA according to the manufacture`s protocol. Unreacted LabelIT Tracker reagent was removed using ethanol precipitation. According to the manufacturer 1 ul of labeling dye per 1 μg plasmid DNA will yield labeling efficiencies of approximately one Label molecule every 40 base pairs (on average) of double-stranded DNA. The purity and concentration of labeled DNA was determined by spectrophotometry.

### Transfection reagents

The following commercially available transfection reagents were purchased: TransIT-X2 (Mirus, Madison, WI), Jet Prime (Polyplus, France), Lipofectamine 2000 (Thermo Fisher, Waltham, MA) and Fugene HD (Promega, Madison, WI). All reagents were used at recommended reagent to DNA ratio to transfect a constant amount of 1 μg DNA for comparison.

### Chemical-based transfection conditions

For transfection experiments, 3x10^5^ 293T cells passaged less than 20 times were plated into 12 well plates the day before transfection. At this density, cells were attached to the well bottom after 24 h at a confluency of 70%–80%. Twenty-four hours after seeding, cells were transfected with FITC-labeled and unlabeled pNL4-3 or pUltraHot encoding mCherry. The transfection was performed using 1 μg plasmid DNA and 3 μl TransIT X2 in serum free media per well, according to the manufactures`protocol. Cells were cultured in complete media. Transfection complexes were removed after 6 hours by replacing all of the media in the well. For time course experiments, the cells were transfected at the same time and harvested at 0, 6, 12, 24, 36, 48 and 72hrs. For experiments comparing different transfection reagents, the cells were transfected with 1 μg DNA/well with varying amounts of transfection reagents to facilitate the recommended ratio of transfection reagent to DNA for each reagent: 3 μl TransIT X2, 4 μl Lipofectamine 2000, 2 μl Jet Prime and 3 μl Fugene HD. Cells were harvested at 24 hrs post-transfection and stained with a viability dye and for intracellular target protein. All experiments were performed in triplicates.

### Electroporation-based transfection

Jurkat E6-1 cells were washed 2 times in OptiMem (Thermo Fisher, Waltham, MA) (without antibiotics) before being resuspended in OptiMem at 1x10^6 cells/ 100 μl. Cells were transferred into 4mm cuvettes (Gene Pulser cuvettes, Biorad, Hercules, CA) and 4 μg of labeled or unlabeled pUltraHot plasmid were added to the suspension. Jurkat E6-1 cells were electroporated with a BioRad Gene Pulser Xcell electroporation system using the exponential protocol with 250 volts, 350uF capacitance, 1000 ohm Resistance. Cells were transferred immediately into pre-warmed media and incubated for 24 hours before staining for viability and flow cytometric analysis.

### Flow cytometry reagents

Anti-HIV-1 p24 monoclonal antibody (71–31) was obtained through the NIH AIDS reagent program #530 and was conjugated with a CF647 dye (Mix-n-Stain Kit from Biotium, Hayward, CA). The matching human IgG1 control was purchased from Biolegend (San Diego, CA) and conjugated with a CF647 dye (Mix-n-Stain Kit from Biotium, Hayward, CA). Amine reactive (Ghost 780 Live/dead dye) and nucleic acid dye (7-AAD: 7-Aminoactinomycin D) were obtained from Tonbo Biosciences (San Diego, CA).

### Fluorescence cell staining for flow cytometry

For flow cytometry, 293T cells were harvested by detaching and washing once in PBS. Cells were fixed in 2% PFA for 15 min and permeabilized in 0.2% Tween/PBS for 15 min. Cells were then stained for HIV-1 p24 protein with 1 μg anti-p24 antibody conjugated to CF647 in 100 μl staining medium containing PBS/2% BSA for 30 min at 4 C, washed and fixed in 1% PFA. Cells transfected with pUltraHot were stained with 1 μl Ghost Violet 450 Live/dead dye in 1 mL PBS. The cells were then washed with 1% FBS in PBS and fixed in 2% PFA for 15 min.

### Flow cytometry analysis

**Fig 1:**
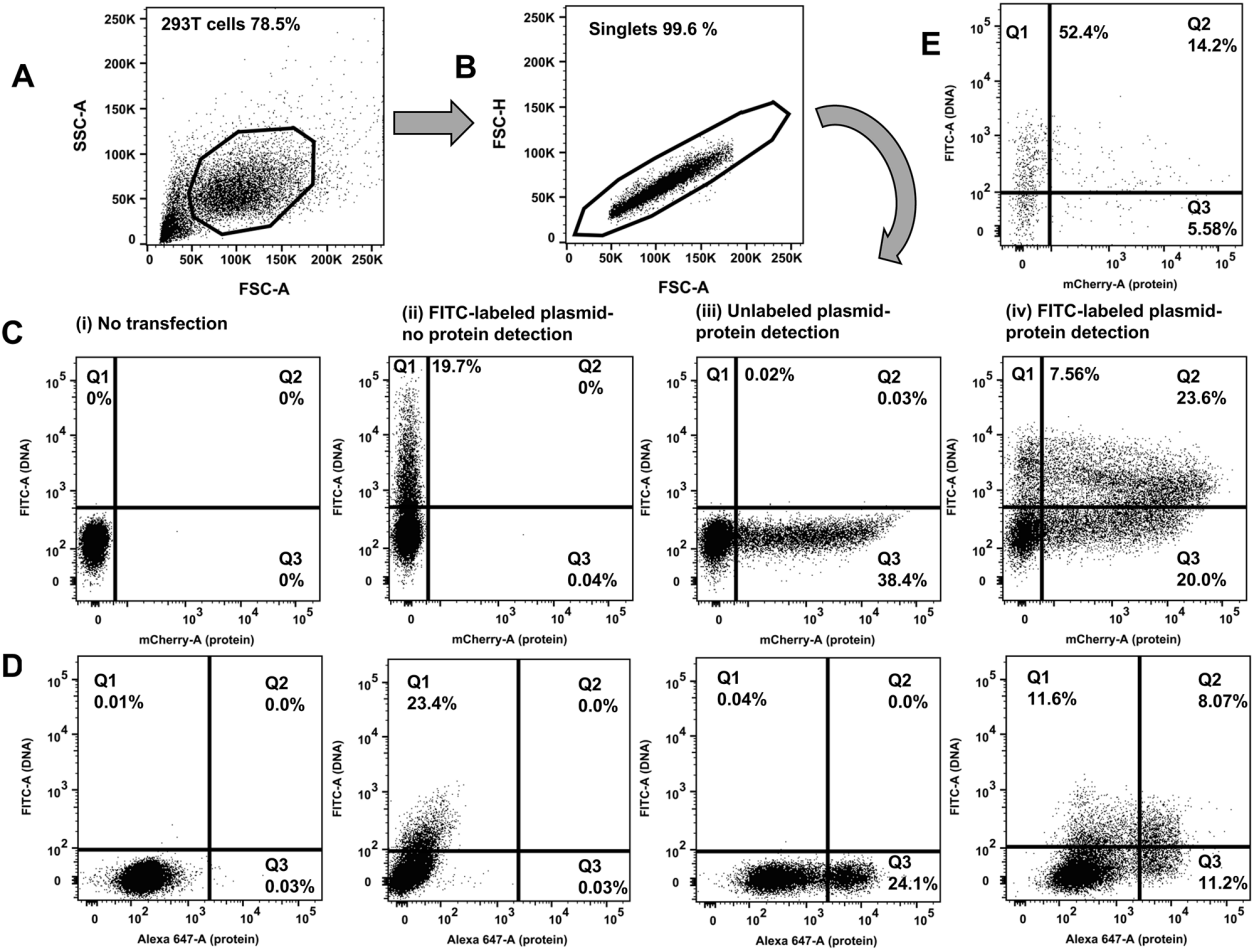
Flow cytometric determination of transfection efficiency based on two independent readouts (DNA plasmid uptake and protein expression). Representative transfections are shown. 293T cells underwent chemical transfection using the TransITX2 transfection reagent as described in Methods. The same amount (1 μg) of DNA was used for two independent plasmids: a small (B: pUltraHot expressing mCherry, 8.3 kb) and a large (C: pNL4-3 expressing p24, 14.0 kb) DNA plasmid. Gating strategy is shown: A) forward and side scatter B) discrimination of doublets C, D) two independent readouts of transfection efficiency. FITC fluorescence corresponds to the uptake of FITC-labeled plasmid DNA (y-axis). A fluorochrome that has no spectral overlap with FITC is used to quantify protein expression. Either a fluorescent protein can be used (e.g. mCherry; shown in C) or a protein labeled with a fluorescent-labeled antibody (e.g. intracellular expression of HIV-1 p24 protein was detected by an CF647-labeled anti-p24 antibody; shown in D). Co-expression of DNA taken up by cells and target protein were analyzed 24 h after transfection. The numbers in the quadrants indicate the percentages of viable cells that took up the FITC labeled DNA plasmid versus the expressed protein that was detected. The following dot plots are shown for each chemical transfection in 293T cells: i) untransfected cells (negative control), ii) cells transfected with FITC-labeled DNA plasmid harvested before protein expression occurred (3 hours post transfection), iii) cells transfected with unlabeled plasmid harvested 24 hours after transfection (when protein expression can be quantified) iv) cells transfected with FITC-labeled DNA plasmid and harvested 24 hours after transfection (when protein expression can be quantified). In this plot Q3 quadrant demonstrates many cells that express protein but do not show any fluorescence associated with uptake of the plasmid DNA. This may reflect effects of the cellular machinery on FITC fluorescence (see Discussion). Either Q1+Q2 (DNA signal) or Q2+Q3 (protein signal) should be used as readouts of transfection efficiency. E. Transfection efficiency was quantified in human lymphocytes (Jurkat E6 cells) harvested 24 hours after electroporation with FITC-labeled DNA mCherry plasmid without the need to use co-transfection of 2 different plasmids and GFP reporter.

Samples were acquired on a LSRII Fortessa flow cytometer (BD Biosciences, San Jose, CA, USA) with BDFACSDiVa^™^ Software (BD Biosciences). The flow cytometer was equipped with 405, 488, 561 and 635 nm lasers, and emission filters for Pacific blue (LP: −, BP:450/50), Alexa fluor-488 (LP: 505, BP: 530/30), PE (LP: −, BP: 582/15), mCherry (LP: 600 BP: 610/20), PerCP-Cy5.5 (LP: 685, BP: 695/40), APC (LP: −, BP: 670/14). The cytometer was routinely calibrated with BD cytometer setup and tracking beads (BD Biosciences). Gating was conducted as shown in Fig 1: First, single cells were gated based on forward scatter area (FSC-A) and forward scatter height (FSC-H) properties, followed by intact cells based on side scatter area (SSC-A) and FSC-A. Uniform cell types (293T cells versus Jurkat T cells) were identified based on their FSC-A and SSC-A scatter properties. Using forward and side scatter parameters, doublet discrimination and the viability stain, dead cells and debris were eliminated from the analysis. Cell viability at 24 and 48 hours was expressed as % cell death based on the death dye and was compared to the viability from the non-transfected control cells. DNA delivery/uptake into cells was determined by Flow cytometric analysis of DNA FITC positive cells and expressed as % of all living cells post-transfection. Protein expression (intracellular HIV-1 p24 or mCherry), was determined by Flow cytometric analysis of A647 or mCherry positive cells. Values are presented as median relative fluorescence intensity (MFI) units. Since incomplete compensation can affect quantification of transfection efficiency when flow cytometry is used, we avoided use of fluorochromes with spectral overlap (1). FITC fluorescence intensity (*y*-axis) is plotted on a log scale against the fluorescence intensity (*x*-axis) of fluorochrome that is used to quantify protein expression (e.g. mCherry) (Fig 1). Background activity of the FITC fluorescence is measured in cells expressing only the transfected unlabeled plasmid and is subtracted from the FITC fluorescence of each transfected sample. Background activity of the fluorochrome that is used to quantify protein expression (e.g. mCherry) is measured at zero timepoint (before any protein is expressed) in cells expressing the unlabeled plasmid. All MFI measurements in transfected cells are blank-corrected with respective MFI values from the transfected cells that are used as negative control (e.g. MFI FITC in transfected minus MFI FITC values in cells transfected with unlabeled plasmid). The MFIs of FITC and A647 can be further standardized using Quantum MESF (Molecules of Equivalent Soluble Fluorochrome) kits for A488 and A647 (Bangs Laboratories, Fishers, IN) as previously described [21]. The fluorescent MESF (Molecules of Equivalent Soluble Fluorochrome) beads contain five different populations of beads, each labelled with a known number of fluorochrome molecules [21]. When this bead mixture is analyzed, the mean fluorescence intensity (MFI) values for each bead peak corresponds to the approximate number of fluorescence molecules associated with a cell in the same MFI range [21]. The FITC and APC channel can be calibrated with these MESF beads on a logarithmic scale, giving a standard curve for MESF values, and by extension FITC and APC molar concentration. Determining the MFI value for each bead peak in the FITC and APC channel allows for the determination of the equivalent MESF values associated with each sample. With the amount of FITC conjugated to the plasmid being a known value, the amount of DNA plasmid can then be calculated at the molar level.

### Statistical analysis

Experiments were performed in three independent cell culture preparations (e.g., biological replicates) unless stated otherwise in the figures. Differences of MFI between different transfection variations were analyzed by two-way analysis of variance (ANOVA) or by Student's *t*-test, or non-parametric equivalent as appropriate. Correlations were assessed with the Spearman rank correlation coefficients. Statistical analyses were performed using GraphPad Prism. Significant differences were accepted when *p*≤0.05.

## Results

### Simultaneous quantification of nucleic acid uptake and protein expression by flow cytometry

**Fig 2:**
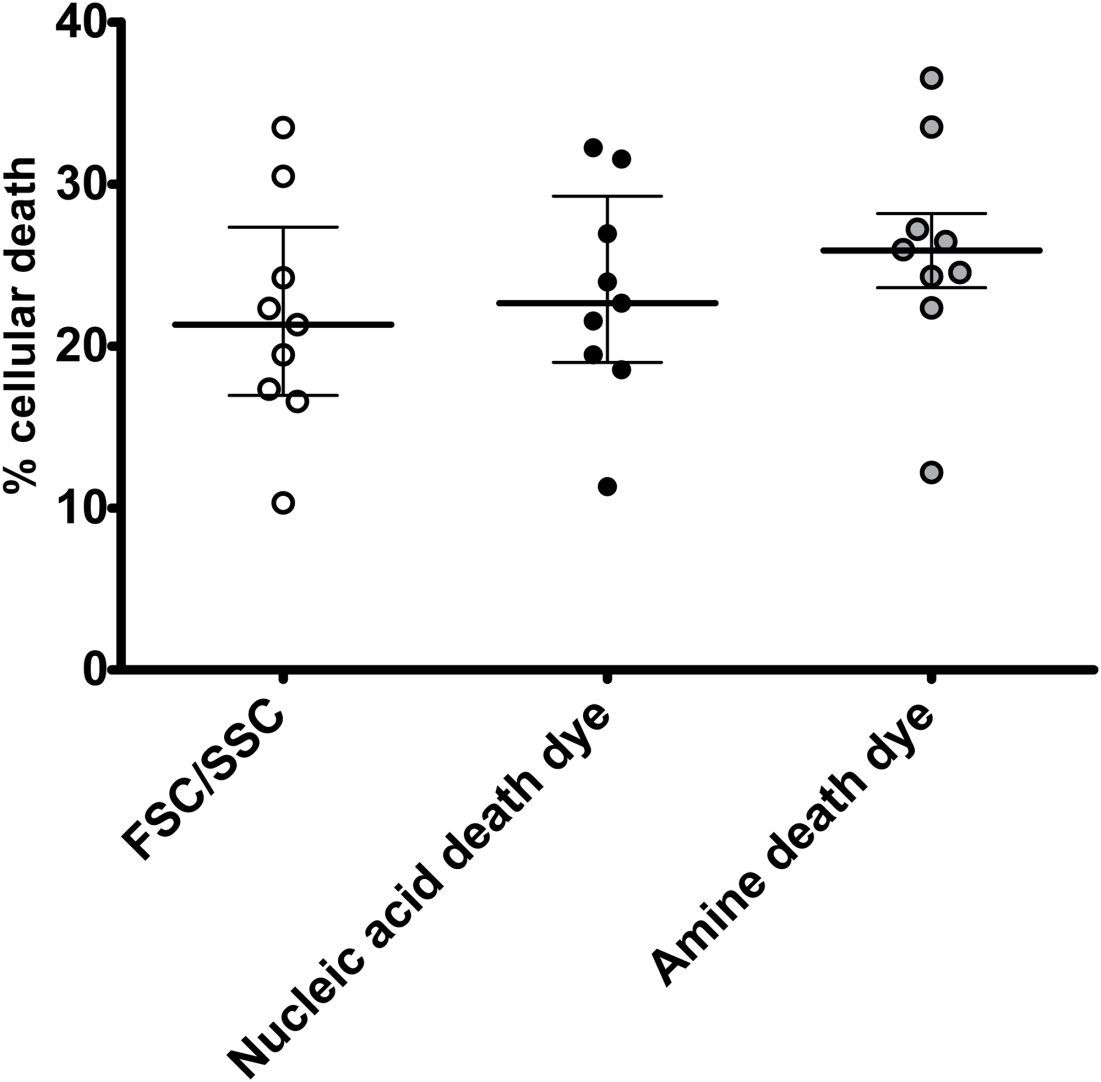
Labeling of the transfected nucleic acids did not affect their function. 293T cells underwent chemical transfection using labeled or standard transfected nucleic acids (pNL4-3 or mCherry plasmids) and the TransITX2 transfection reagent as described in Methods. Jurkat E6 cells underwent electroporation with FITC-labeled DNA mCherry plasmid as described in Methods. Each experiment was performed in triplicates and three independent times with specific (un)labelled nucleic acids. Viable cells were gated based on either forward and side scatter (FSC/SSC) or viability dye as shown in Fig 3 and transfection efficiency was determined by comparing protein expression compared to untransfected control. Data in each experiment were normalized by the average of the experimental control (standard transfected nucleic acids) in each experiment and were then pooled together. This approach increases statistical power while taking into consideration the inherent differences in transfection efficiency among different transfection methods (chemical transfection versus electroporation in 293T cells versus Jurkat E6 cells). The non-parametric statistical Kruskal-Wallis test was used for comparisons between the labeled and unlabeled transfected nucleic acids. Median and interquartile range (IQR) are shown. The use of unlabeled transfected nucleic acids (standard transfection method) lead to similar protein expression of target gene [100, (14)] compared to the labeled transfected nucleic acids, regardless of the method of viability gating (FSC/SSC; 94.5, (20) vs viability dye; 101 (16)] (p = 0.745).

To develop a robust flow cytometric method that can be used to reliably quantify transfection efficiency at the single cell level and to avoid limitations of GFP-based flow cytometric methods that can compromise accuracy of determination of transfection efficiency [6, 9–15], we developed a flow cytometric method that is based on two independent readouts of transfection efficiency and on direct labeling of the transfected plasmid DNA. The FITC fluorochrome is used to detect intracellular levels of the transfected plasmid that has been labeled with FITC (Label IT tracker)(Fig 1A–1E). The second fluorochrome is used to quantify expression of the target protein [by directly measuring fluorescence of the expressed protein if the target protein is fluorescent e.g. mCherry (Fig 1C) or by using a fluorescent-labeled antibody against the target protein (Fig 1D)]. Fig 1 shows the gating strategy of the flow cytometric method. To develop and optimize this method, we focused on the 293T cell line that is known to be an excellent cell line for quantification of transfection efficiency of various plasmids. Of note the labeling of the transfected nucleic acids did not affect their function, i.e. transcription since similar amounts of target protein were detected with use of labelled or unlabeled (standard method) plasmids (Figs 1 and 2).

However, our novel flow cytometric method can also be used as a tool to optimize transfection efficiency in difficult to transfect human cells without the need to co-transfect a GFP reporter plasmid that can further compromise transfection efficiency (Fig 1D). We validated our method using two independent labeled plasmids, the pNL4-3 plasmid (14 kB) that encodes the entire HIV genome and the smaller (8.3 kB) pUltraHot plasmid that encodes for the mCherry fluorescent protein. Using this method both plasmid DNA and protein can be quantified simultaneously in the same tube at the single cell level, while gating in viable cells.

### The confounding effect of cellular toxicity on determination of transfection efficiency can be minimized with the novel flow cytometric method

**Fig 3:**
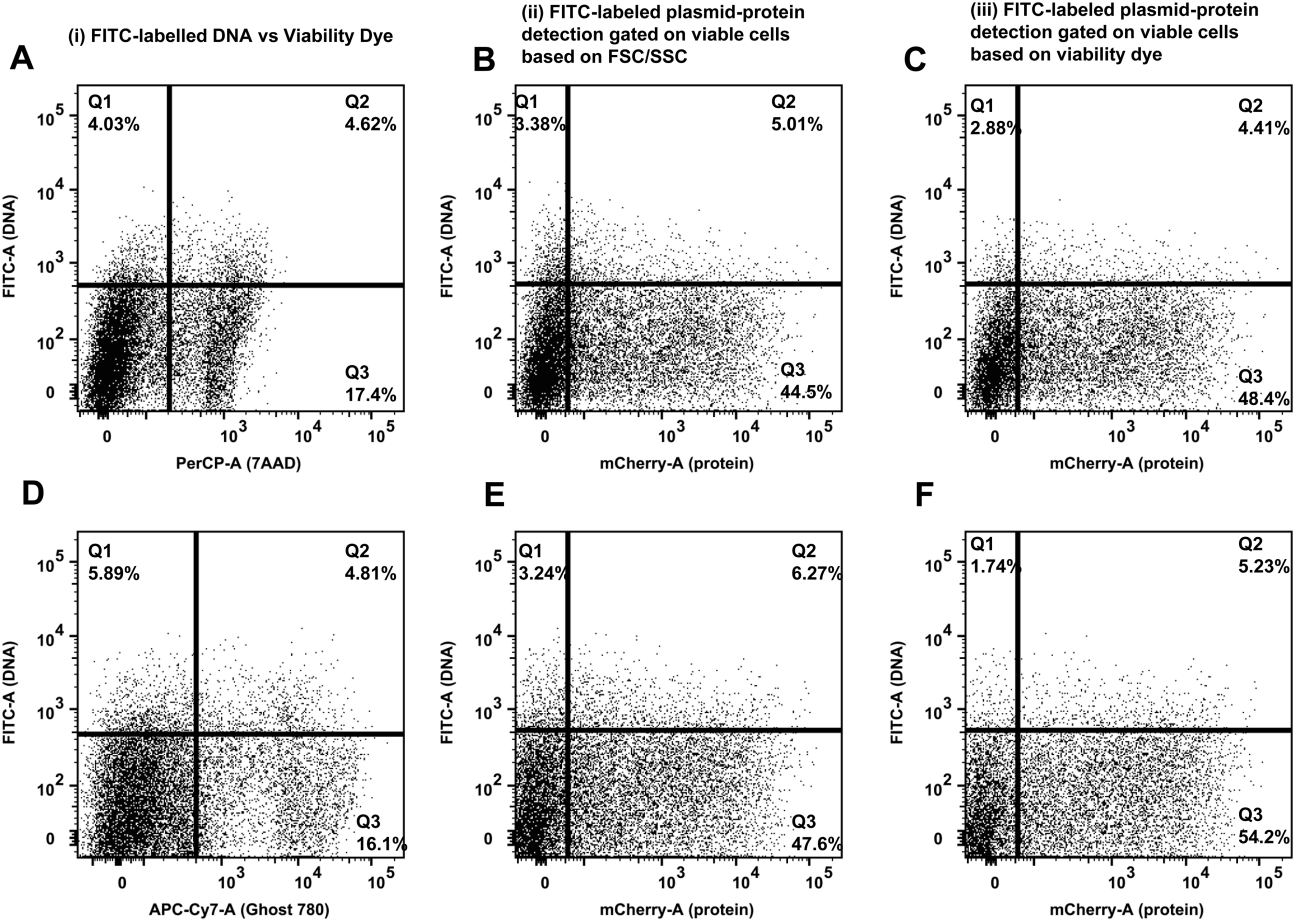
Determination of cell viability in described flow cytometric method that quantifies transfection efficiency. Representative transfections are shown. 293T cells underwent chemical transfection using the TransITX2 transfection reagent and the mCherry plasmid as described in Fig 1. Cell viability and co-expression of DNA taken up by cells and target protein were analyzed 24 h after transfection. Cell viability was assessed by flow cytometry using two independent approaches: a) viable cells were gated based on forward and side scatter as shown in Fig 1 b) viable cells were gated based on nucleic acid [7-AAD: 7-Aminoactinomycin D, A-C] and amine reactive (Ghost 780 Live/dead dye, D-F) viability dyes. Representative plots from 3 independent experiments are shown. As expected the chemical transfection and the DNA plasmids per se are toxic to the cells. Similar data were obtained using 2 independent viability dyes [7AAD (A), Ghost 780 (D)]. Overall cellular toxicity was similar (10–40%) among the 3 methods [FSC/SSC (Fig 1), nucleic acid and amine reactive death dyes](Fig 4). The mean viability of the untransfected 293T cells was >85%. In addition, determination of the transfection efficiency based on two independent readouts (DNA plasmid uptake and protein expression) was similar by gating on viable cells based on either FSC/SSC (B, E) or viability dye (C, F).

**Fig 4:**
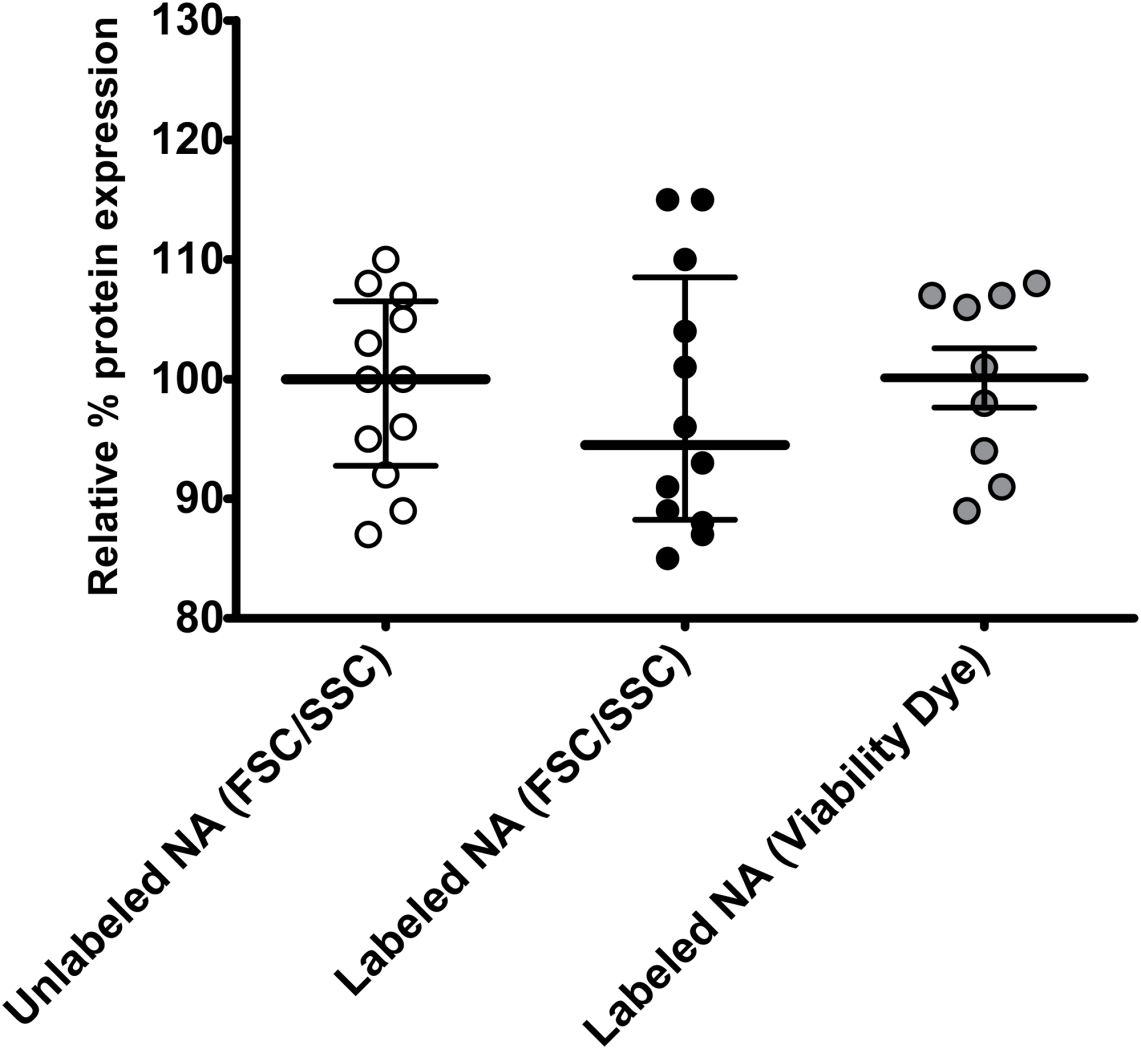
Cellular toxicity can be determined in described flow cytometric method that quantifies transfection efficiency without the use of a viability dye. 293T cells underwent chemical transfection using labeled or standard transfected nucleic acids (pNL4-3 or mCherry plasmids) and the TransITX2 transfection reagent as described in Methods. Gating on viable cells was performed as shown in Fig 1. Each experiment was performed in triplicates and three independent times with specific (un)labelled nucleic acids. Median and interquartile range (IQR) are shown. The non-parametric statistical Kruskal-Wallis test was used for comparisons between groups. Quantification of cell death by forward and side scatter parameters, and doublet discrimination [18.4% (8.6)] gave similar results compared to quantification of cell death by nucleic acid viability dye (7-AAD) [20.50% (11.7)] and amine viability dye [24.4 (11.1)](p = 0.379). Of note use of viability cell dye cannot be used in electroporation experiments (e.g. Jurkat E6 cells electroporation with FITC-labeled DNA mCherry plasmid) due to the mechanisms of action of electroporation methods and dye exclusion tests for cell viability dyes.

Toxicity of DNA and the transfection method per se should also be taken into consideration during transfection [22] but most standard methods that are used to quantify transfection efficiency (such as the luciferase method) do not take this important variable into consideration. A third fluorochrome can be used to quantify cellular toxicity (death) as a direct result of the transfection (Fig 3A and 3D). The direct toxicity as a result of the DNA plasmid and transfection reagent can be quantified by the percentage of cells expressing the FITC labeled DNA plasmid that are also positive for the death dye detected in an appropriate channel (Fig 3A and 3D). Alternatively, the confounding effect of cellular toxicity can also be minimized without use of a death dye since quantification of cell death by forward and side scatter parameters, and doublet discrimination gave similar results compared to quantification of cell death by nucleic acid (7-AAD) or amine viability dye (Figs 3 and 4). In addition, determination of the transfection efficiency was similar by gating on viable cells based on either FSC/SSC or viability dye (Figs 2 and 3). Since viability dyes cannot be used in certain experiments such as electroporation, gating on viable cells based on FSC/SSC is a reasonable approach. Thus, the novel flow cytometric method can be used to limit analysis of transfection efficiency to viable cells and can minimize the effects of direct cellular toxicity of transfection on the determination of transfection efficiency.

### The novel flow cytometric method that quantifies transfection efficiency is reproducible

**Fig 5:**
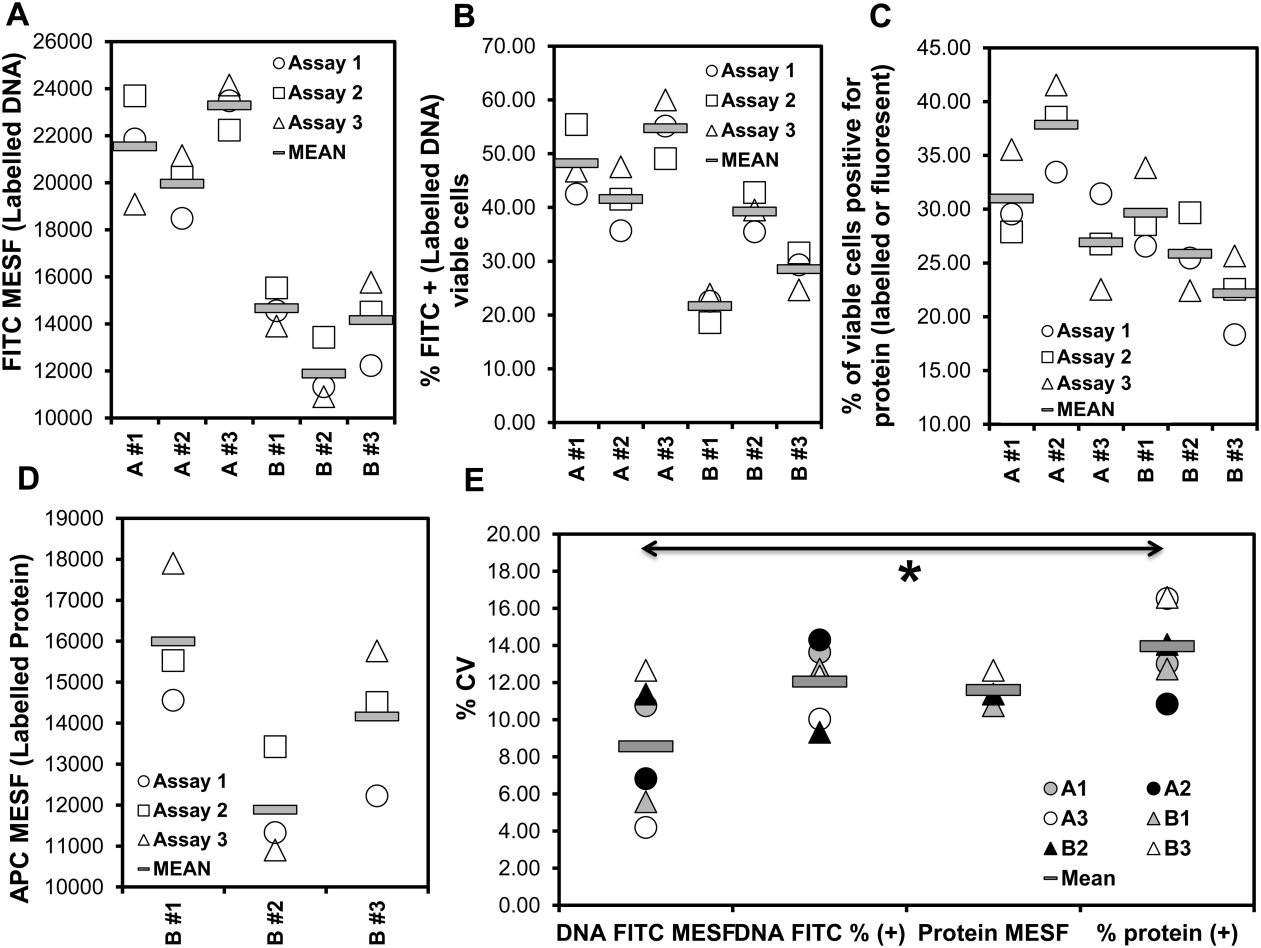
Standardization and reproducibility of described flow cytometric method that quantifies transfection efficiency. Three different stocks of 293T cells (1–3,<20 passages) underwent chemical transfection using the TransITX2 transfection reagent and the mCherry plasmid (plasmid A) and the NL4.3 plasmid (plasmid B) as described in Fig 1. Co-expression of DNA taken up by cells and target protein were analyzed 24 h after transfection. MESF (Molecules of Equivalent Soluble Fluorochrome) beads were used to standardize median fluorescence intensity (MFI) units as described in Methods. The four measures of transfection efficiency [A: MESF of FITC (labeled DNA) in viable cells, B: % FITC+ (labeled DNA) of viable cells, C: % viable cells positive for protein (labelled or fluorescent), D: MESF of fluorochrome used to label protein (APC in this case) in viable cells] are means of triplicates from three independent experiments (Assay 1–3) and are plotted in A-D. Note that MESF beads are not available for mCherry and in this case the MFI can be used to quantify levels of expression of protein per cell. The comparison of the coefficient of variation (CV%) among the 4 independent readouts is shown in E. The mean inter-assay variability for these six samples (A1-3, B1-3) for the different readouts (A-D) was as follows: A: 8.56% (range 4.19 to 12.66%), B: 12.06% (range 9.32 to 14.30%), C: 11.59% (range 10.75 to 12.66%), D: 13.96% (range 10.84 to 16.59%). The readout A was more reproducible (p<0.05, ANOVA). Similar standardization can be established with various cells and transfection methods (e.g. Jurkat E6 cells electroporation with FITC-labeled DNA mCherry plasmid).

Using the approach described in Fig 1 and standardized fluorescence units (MESF) beads, the transfection efficiency for a specific target can be standardized using two independent readouts (amount of DNA uptake and detected expressed protein) (Fig 5). Overall, the MESF of FITC (labeled DNA) in viable cells was the most reproducible readout of transfection efficiency (mean interassay coefficient of variation 8.56%, range 4.19 to 12.66%)(Fig 5). Thus, the standardized fluorescence per cell provides a more reproducible measure of transfection efficiency compared to the percentage of transfected cells in a cell population.

### Optimization of timing to determine transfection efficiency using the novel flow cytometric method

**Fig 6:**
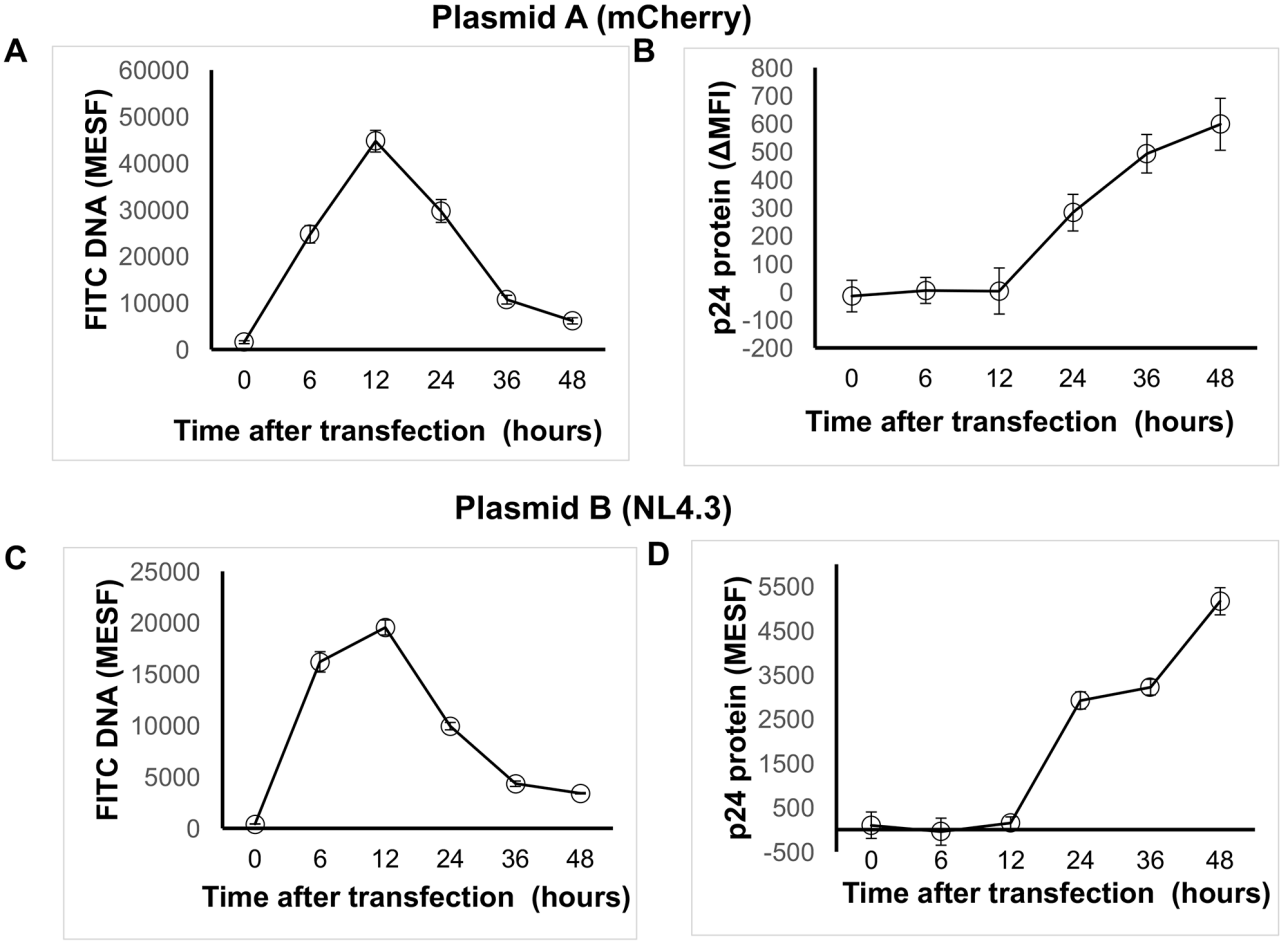
Flow cytometric analysis of DNA uptake and protein expression over time in described flow cytometric method that quantifies transfection efficiency. 293T cells (<20 passages) underwent chemical transfection using the TransITX2 transfection reagent and the mCherry plasmid (plasmid A; A, B) or the NL4.3 plasmid (plasmid B; C, D) as described in Fig 1. Co-expression of DNA taken up by cells (A, C) and target protein (B, D) were analyzed at 6, 12, 24, 36 and 48 hours after transfection. Data are means of triplicates from three independent experiments. Median fluorescence intensity was subtracted from the respective untransfected control (ΔMFI) as described in Methods. Fluorescence intensities were standardized using a MESF standard curve as described in methods. Note that MESF beads are not available for mCherry and in this case the MFI can be used to quantify levels of expression of protein per cell.

There is limited published evidence regarding the optimal timing after transfection for determination of transfection efficiency [9, 10, 22]. Using the methodology described in Fig 1 we found that the peak uptake of two independent labeled plasmids occurred after 6 hours after transfection and that the peak protein expression was evident within 48 hours (Fig 6). For both plasmids, the peak uptake of plasmid DNA (FITC) was seen at 12 hours. A linear reduction in the DNA FITC signal with a simultaneous increase in the protein signal was seen after 12 hours. Similar optimization of timing to determine transfection efficiency can be established with various cells and transfection methods (e.g. Jurkat E6 cells electroporation with FITC-labeled DNA mCherry plasmid). Thus, the current method can be used as a tool to optimize the timing of determination of transfection efficiency, but specific time-course experiments need to be performed for specific plasmid and transfection reagents/methods.

### The novel flow cytometric method can be used as a tool to optimize transfection by selecting the best for a specific type and amount of plasmid transfection reagent

**Fig 7:**
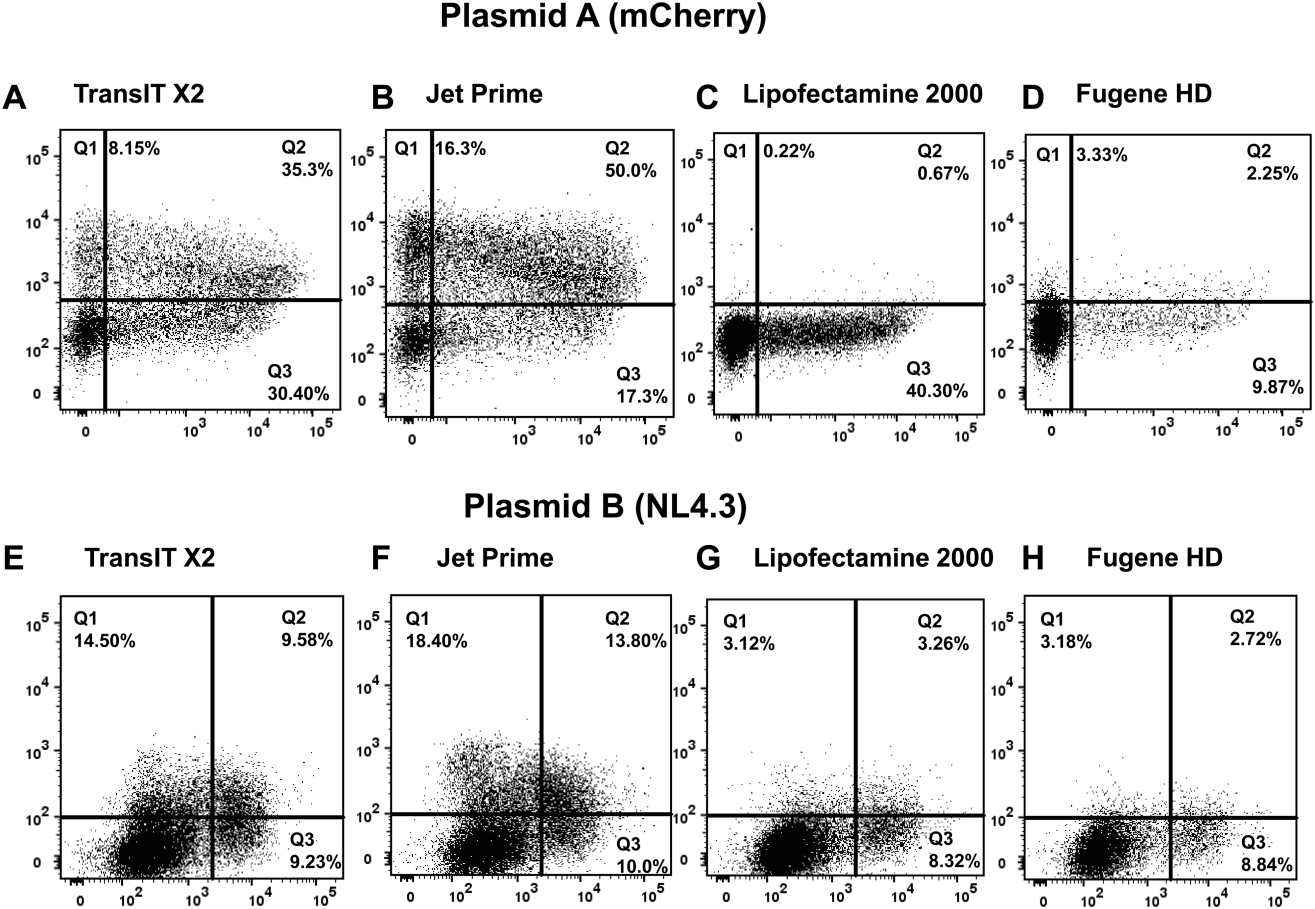
Comparison of major commercially available transfection reagents using described flow cytometric method that quantifies transfection efficiency. 293T cells underwent chemical transfection using different commercially available transfection reagents and the mCherry plasmid (plasmid A; A, B, C, D) or the NL4.3 plasmid (plasmid B; E, F, G, H) as described in Fig 1. Co-expression of DNA taken up by cells and target protein were analyzed at 24 hours after transfection. Data are means of triplicates from three independent experiments. For each reagent the ratio of transfection reagent to DNA amount was optimized as per manufacturer`s instructions and the same (1 μg) amount of DNA was used.

**Fig 8:**
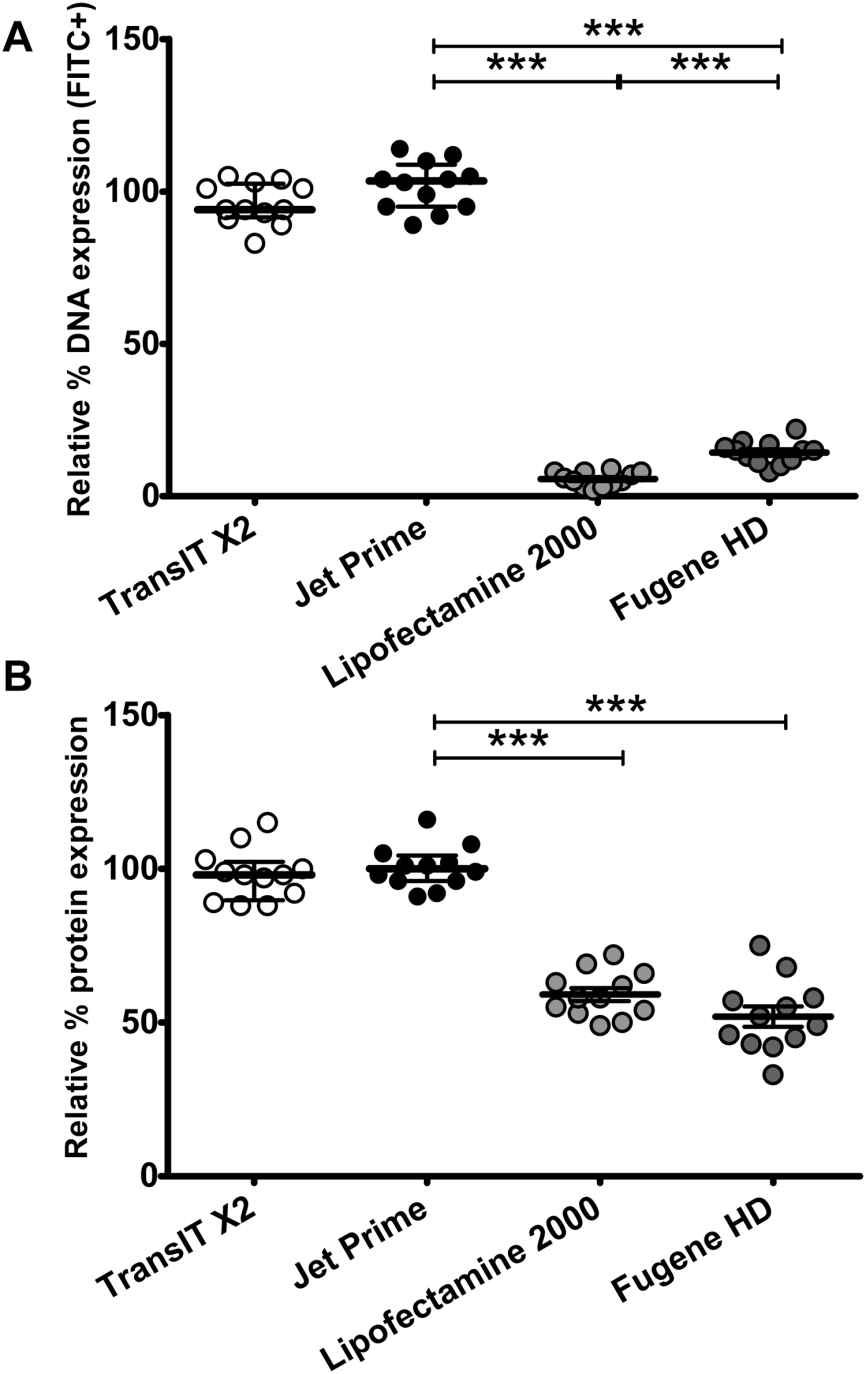
Comparison of major commercially available transfection reagents using described flow cytometric method that quantifies transfection efficiency. 293T cells underwent chemical transfection using labeled or standard transfected nucleic acids (pNL4-3 or mCherry plasmids) and different commercially available transfection reagents as described in Methods. Gating on viable cells was performed as shown in Fig 1. Each experiment was performed in triplicates and six independent times (6 per plasmid and 12 in total) with specific (un)labelled nucleic acids. Data in each experiment were normalized by the average of the experimental control (standard transfected nucleic acids) in each experiment and were then pooled together. This approach increases statistical power while taking into consideration the inherent differences in transfection efficiency among different transfection methods (different chemical transfection reagents and plasmids). The non-parametric statistical Mann-Whitney test was used for comparisons between groups. Median and interquartile range (IQR) are shown. The transfection efficiency was assessed by both amount of labelled DNA (A) and protein (B). The use of TransIT-X2 and Jet Prime gave the best (and comparable) transfection efficiencies, whereas the efficiency was reduced with Lipofectamine 2000 and Fugene HD. There was a major decrease in the amount of detectable labelled DNA with Lipofectamine 2000 and Fugene HD.

To further optimize transfection efficiency, we directly compared four major commercially available nonviral reagents of chemical transfection using the same approach as in Fig 1 and the best time point (24 hours as determined in Fig 6) for the use of both independent readouts (labeled plasmid and expressed protein) of transfection efficiency. For each transfection reagent, we chose the optimal ratio of transfection reagent to the amount of DNA plasmid that produced the highest FITC signal based on recommendations from the manufacturer. Using two independent plasmids, TransIT-X2 and Jet Prime gave the best (and comparable) transfection efficiencies, whereas the efficiency was reduced with Lipofectamine 2000 and Fugene HD (Figs 7 and 8). We also determined the viability of the cells during the chemical transfection with different reagents. Using the approach described in Figs 1 and 2 and death dye, all transfection reagents had similar cellular toxicity at 24 hours after transfection (overall cell death 8.2–12.6%). Thus, using our flow cytometric method for a specific amount of a certain plasmid, different transfection reagents can be directly compared with regards to efficiency and cellular toxicity and thus the efficiency of chemical transfection can be further optimized.

### Using the novel flow cytometric method, transfection efficiency can be reliably quantified as early as 6 hours after transfection

**Fig 9:**
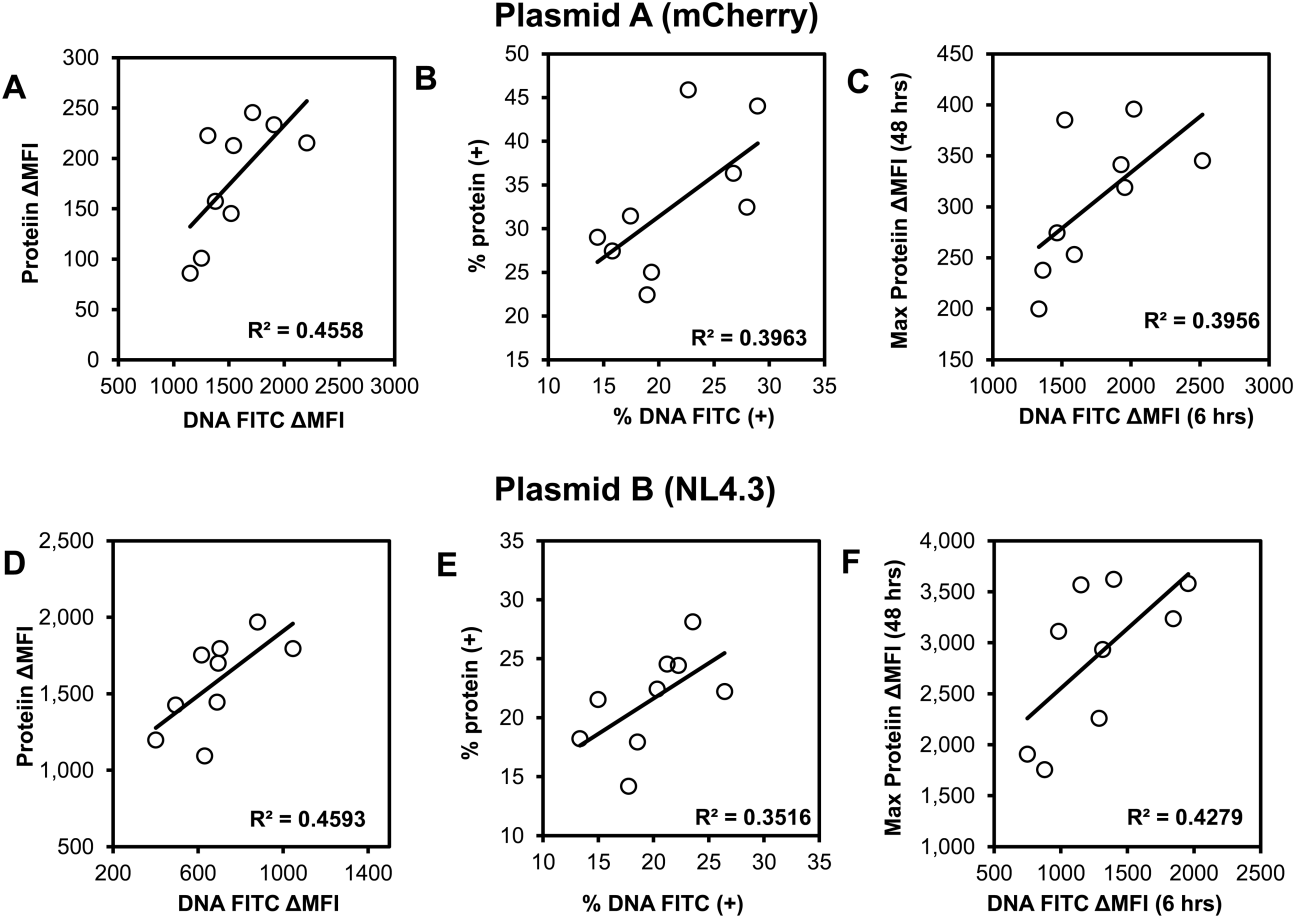
Correlations between readouts of described flow cytometric method that quantifies transfection efficiency. 293T cells (<20 passages) underwent chemical transfection using the TransITX2 transfection reagent and the mCherry plasmid (plasmid A, A-C) or the NL4.3 plasmid (plasmid B, D-F) as described in Fig 1. Co-expression of DNA taken up by cells and target protein were analyzed at 6 (C,F), 24 (A,B, D, E) and 48 (C, F) h after transfection. Median fluorescence intensity was subtracted from the respective untransfected control (ΔMFI) as described in Methods. Spearman correlations between the same readouts (% positive of viable cells vs ΔMFI in viable cells) are shown for labeled DNA vs labeled protein for the two different plasmids. Overall, ΔMFIs correlated better than % positivity of viable cells (all p values≤0.05). In addition an early (6 hours) readout from labeled DNA (FITC) correlated significantly with the maximum (48 hours) ΔMFI that corresponded to expression of protein per cell (C, F).

The MESF readouts (at 24 hours after transfection) for labeled plasmid and expressed protein, correlated better compared to the % positivity for labeled plasmid and expressed protein (Fig 9). In addition an early (6 hours) readout from labeled DNA (FITC) correlated significantly (r = 0.65–0.8, p<0.05) with the maximum (48 hours) median fluorescence per cell that corresponded to expression of protein per cell. Thus, our method is rapid and can reliably be used to quantify transfection efficiency as early as 6 hours after transfection.

## Discussion

Here we provide a relatively simple, rapid and robust flow cytometric method that can be used as a tool to optimize transfection efficiency at the single-cell level. This method takes into consideration cell toxicity as a direct result of the transfection and the nucleic acid per se and uses two independent readouts of transfection efficiency: a) the amount of plasmid nucleic acid that cells have taken up during transfection b) the amount of the encoded expressed protein. It is important to emphasize that any nucleic acid plasmid can be labeled with the LabelIT Tracker, making this method very versatile and applicable to any transfection method. We observed a higher transfection efficiency based on DNA FITC signal and protein expression with a smaller plasmid, which is in line with the nature of large plasmid requiring more transfection reagent to complex the DNA and being more difficult to be transported across the plasma membrane than smaller constructs. We here also discuss important considerations required to successfully implement this method that can be used to optimize several variables that can affect transfection efficiency such as cellular toxicity related to the transfection method, the time of exposure of a standard cell line to specific nucleic acid plasmid and the type of transfection reagent. This method overcomes limitations of prior established methods such as dual reporter systems [6, 9–15] and measures reliably the efficiency of transfection into human cells and cell lines.

Transfection is an important tool in molecular biology that is applicable for gene therapy studies in humans using methods such as LTR-Specific Tre-Recombinase [23], the Sleeping Beauty (SB) transposon system [24] and the clustered, regularly interspaced, short palindromic repeat (CRISPR) technology [25]. One of the biggest challenges to investigate the protein function and interaction in gene therapy studies is to have reliable monospecific detection reagents, particularly antibodies, for all human gene products [26, 27]. Transfection efficiency can be affected by multiple experimental parameters such as the choice of the transfection method, health, and viability of the cell line, degree of confluency, quality and quantity of the nucleic acid used [1, 2]. The ideal approach should be selected depending on specific cell type and experimental needs, and should have high transfection efficiency, low cell toxicity, minimal effects on normal physiology, and be easy to use and reproducible [1, 2]. Thus, a rapid, robust and reliable method that can be used to efficiently quantify transfection efficiency based on not only expression of the target protein of interest but also the uptake of the nucleic acid plasmid, can be an important tool in molecular biology.

The measure of transfection efficiency, the percentage of transfected cells in a cell population, is a subjective measure prone to many variable factors, such as cell cycle progression, the circadian rhythm of gene expression activity, promoter activity, and general activity of a given cell type. All of these factors can inhibit a cell from actually expressing the transfected protein. Commonly used methods to quantify transfection efficiency such as the luciferase assay, require cell lysis, do not quantify overall cell toxicity, and thus the determination of the percentage of transfected cells in a cell population may not be accurate [4]. Indeed, in prior work using luciferase reporter constructs to compare different transfection reagents, arbitrary units per protein content were used as readout instead of a percentage of transfected cells [28]. On the other hand, flow cytometric methods to quantify transfection efficiency in cells based on transfection of GFP-fusion proteins or co-transfection of GFP plasmids have limitations including competition in expression of the two different plasmids [9] and artifacts of GFP fluorescence during processing of cells or tissues [6, 9–15]. Maeβ et al reported fluorescence labelling of siRNA in the setting of transfection of macrophages but did not clarify details of the labelling method of the nucleic acid (siRNA), the method was not standardized and there was no simultaneous measurement of target protein as a tool to quantify transfection efficiency[29]. Herein, we standardized median fluorescence intensity of two independent readouts of transfection efficiency (amount of DNA that cells have taken up during transfection as well as the amount of the expressed target protein) using MESF beads. This approach provides an objective and more reproducible (compared to the percentage of transfected cells in a cell population) measure of transfection efficiency.

We also used this method to demonstrate that it can be used as a tool to detect transfected DNA in difficult to transfect human cells without the need to co-transfect plasmids for reporters that can further compromise transfection efficiency. The use of plasmid vectors for the *ex vivo* genetic modification of human tissue/cell types for therapeutic purposes has been limited by the low efficiency of currently available plasmid transfection systems and their cell toxicity [30–32]. Since lymphocytes are notoriously refractory to most kinds of nonviral DNA delivery methods that have significant toxicity, robust methods to quantify transfection efficiency as well as transfection-induced cellular toxicity are needed. Thus, our method can directly detect labeled transfected nucleic acid in viable cells after electroporation at the single cell without the need to use co-transfection of two different plasmids involving a reporter gene.

One of the limitations of nonviral gene delivery systems is also their toxicity that can significantly compromise transfection efficiency. Other common methods that quantify transfection efficiency such as luciferase assay do not quantify cell toxicity of the transfection method [4]. Our assay can quantify not only the transfection efficiency with 2 different readouts (DNA-FITC and protein) but also the cellular toxicity. Quantification of transfection efficiency at the single cell level is based on viable target cells and this approach minimizes confounders such as the effect of cellular toxicity on overall transfection efficiency and the effect of detachment of adherent cells on cell death rate. Thus depending on the application and the cell type this information can be used to further optimize transfection efficiency.

Incubation time for optimal protein detection after transfection varies (3–48 hours) between different plasmids and transfection methods depending on the protein of interest and its post-translational modifications, location and turnaround time [10]. Due to aforementioned limitations of prior methods to quantify transfection efficiency, there is limited published evidence regarding the optimal timing after transfection for determination of transfection efficiency [9, 22]. Interestingly, we found that the DNA uptake peaked between 6–12 hours after transfection. The low capacity of DNA to escape from endosomes is regarded a major limitation of their transfection efficiency [33]. Thus carriers (such as polymers, peptides and lipids) based on the exploitation of the imidazole ring as an endosome destabilization device after often used with plasmids to enhance nucleic acid delivery in the cytosol [33]. DNA plasmids are also often condensed by protamine to enhance cytosolic delivery[34–36]. Cellular processes related to DNA are governed by the intricate interplay between different states of DNA compaction that often have surprising and poorly described properties [37]. Thus, the DNA plasmid may open later after initial cellular uptake and an unexpected peak in its detection may be seen hours after initial uptake. Our method based on fluorescent labeling of DNA plasmid can be further optimized with the use of novel methods such as ImageStream flow to study in detail cytoplasmic and nuclear transfer process [38]. Thus, with our flow cytometric method cells can be harvested at multiple times throughout an experiment to generate a time-course experiment and optimize the best time to determine peak transfection efficiency.

There is limited published evidence regarding direct comparison of the major commercially available transfection reagents (for the same plasmid and cell type)[22]. With our proposed flow cytometric method, it is possible to compare several different transfection products simultaneously and thereby reduce result variability. Out of the numerous commercially available transfection reagents, we chose four nonviral transfection reagents which can be used in serum-containing media. The first one, Lipofectamine^™^ 2000 (Invitrogen) transfection reagent, is based on the lipofection method [39]. Lipofectamine reagents are often considered the “gold-standard” of transfection reagents and are used as a basis of comparison for efficiencies of other transfection methods [40]. The second transfection reagent evaluated in this study was JetPrime^™^ (Polyplus transfection, France), which is a nonliposomal, polymer-based transfection reagent. Both lipofection and polymer transfections, like JetPEI [41] and JetPrime, involve extensive membrane interaction in the form of liposome fusion or charge binding and subsequent endocytosis, respectively. The third transfection reagent was *Trans*IT-X2^®^ Dynamic Delivery System an advanced non-liposomal system of proprietary polymers that aid in nucleic acid complexation, uptake and endosomal release thereby enabling superior transfection of plasmid DNA and smaller nucleic acids. Finally, the fourth transfection reagent was FuGENE^®^ HD Transfection Reagent a nonliposomal formulation designed to transfect DNA into a wide variety of cell lines with high efficiency and low toxicity. While these four different transfection reagents only represent a small fraction of commercially available products, our flow cytometric assay is a valuable tool for future evaluation of any transfection reagent in specific cell types.

Interestingly, a drastic reduction in the detection of the FITC labeled DNA was seen when Lipofectamine 2000 and Fugene HD were used for chemical transfection. It is possible that quenching of FITC in endosomes/lysosomes may be an explanation for our findings given the nature of the transfection reagents and that FITC is a pH-sensitive fluorescent dye [42, 43]. It is also possible that fluorescence labeling inhibits complex formation/transfection with Lipo/Fugene. In addition, we found that addition of amine reactive death dyes interfered with the FITC readout and gave variable and inconsistent results. Different fluorochromes (CX-Rhodamine, Cy^®^3, Cy^®^5, TM-Rhodamine) that are not as pH-sensitive dyes as FITC can be used to label plasmid nucleic acid (RNA or DNA)[44–46]. The precise mechanisms of how the plasmid DNA interacts with different cellular compartments are not well characterized. Based on our results, we suggest that chemicals (such as TransIT-X2^®^) that are optimized to allow escape of the nucleic acid plasmid from endosomes/lysosomes should be preferred when our flow cytometric method is used to quantify efficiency of chemical transfection. Finally, possible biochemical interactions with individual fluorochromes and the DNA LabelIT Tracker need to be individually tested for each transfection method.

Any nucleic acid (DNA or RNA) plasmid can be labeled with the LabelIT Tracker, making this method very versatile and applicable to any transfection method. This method is applicable to transfection methods that have revolutionized the field of gene therapy such as the bacterial CRISPR/Cas9 system. CRISPR consists of two components: a “guide” RNA (gRNA) and a non-specific CRISPR-associated endonuclease (Cas9) [47–50]. The genomic target of Cas9 can be changed by simply changing the targeting sequence present in the gRNA. Once expressed, the Cas9 protein and the gRNA form a riboprotein complex [47–50]. For some CRISP libraries, Cas9 (or Cas9 derivative) is included on the gRNA-containing plasmid; for others, they must be delivered to the cells separately [47–50]. Mammalian expression vector containing promoters for Cas9 and gRNA, lentiviral transfer vector containing Cas9 and gRNA, RNA plasmids containing gRNA and Cas9 mRNA and purified Cas9 protein-gRNA riboprotein complexes have all been used as major expression systems and variables for using CRISPR in mammalian cells [25, 51–53]. Although genomic cleavage PCR detection methods and sequencing of genomic DNA are ultimately needed to quantify efficiency and accuracy of the CRISP system for a specific application, these methods are not efficient tools to optimize all pertinent experimental variables related to the transfection per se [53–55]. Reporter gene (e.g. GFP) to identify and enrich positive cells, or selection marker to generate stable cell lines have been used but reporter systems have several aforementioned limitations. The approach presented here allows labelling of any nucleic acid and simultaneous detection of target protein and could be adapted to optimize delivery of Cas9/gRNA cassettes for genome editing in a variety of systems. Commercially available monoclonal antibodies targeting the Cas protein (clone 7A9-3A3) are available (Cell Signalling Technology) and can be used for flow cytometry. In addition, different fluorochromes (FITC, CX-Rhodamine, Cy^®^3, Cy^®^5, TM-Rhodamine)[44–46] can be used to simultaneously label both the gRNA and the Cas9 nucleic acid (mRNA or pDNA).

## Conclusion

Taken together, the described relatively simple, rapid and robust flow cytometric method can be used as a tool to optimize transfection efficiency at the single-cell level. This method overcomes limitations of prior established methods (such as dual reporter systems) to quantify transfection efficiency. Any DNA or RNA template can be labelled and is suitable for a wide range of applications. The one-step chemical method is easy and allows consistent control of the labeling reactions and high sensitivity of detection of plasmid DNA can be achieved with optimally labeled DNA and RNA. In addition, the fluorescent labels do not impact hybridization performance and the covalent mechanism allows permanent, non-destructive modification of nucleic acid residues that is ideal for many diverse applications. Given the importance of transfection in molecular biology that has set the basis for gene therapy studies in humans, this novel flow cytometric method can be an important tool in molecular biology and gene therapy studies using methods such as the Sleeping Beauty (SB) transposon system, the CRISPR technology and in cases where reliable monospecific detection methods for human gene products (protein) do not exist.
